# Socio-economic factors and virological suppression among people diagnosed with HIV in the United Kingdom: results from the ASTRA study

**DOI:** 10.7448/IAS.17.4.19533

**Published:** 2014-11-02

**Authors:** Lisa Burch, Colette Smith, Jane Anderson, Lorraine Sherr, Alison Rodger, Rebecca O'Connell, Richard Gilson, Jonathan Elford, Andrew Phillips, Andrew Speakman, Margaret Johnson, Fiona Lampe

**Affiliations:** 1 Department of Infection and Population Health, University College London, London, UK; 2 Centre for the study of Sexual Health and HIV, Homerton University Hospital, London, UK; 3 Department of Sexual Health, Newham University NHS Hospital Trust, Barts Health, London, UK; 4 School of Health Sciences, City University London, London, UK; 5 Department of HIV Medicine, Royal Free NHS London Foundation Trust, London, UK

## Abstract

**Introduction:**

In the United Kingdom, rates of virological suppression on antiretroviral therapy (ART) are very high, but there remain a small but significant number of people on ART with detectable viraemia. The impact of socio-economic factors on virological suppression has been little studied.

**Materials and Methods:**

We used data from ASTRA, a cross-sectional, questionnaire study of >3000 individuals from 8 clinics in the United Kingdom in 2011–2012, linked to clinical records to address this question. Included participants had received ART for >6 months with a recorded current viral load (VL) (latest at the time of questionnaire). Participants provided data on demographic factors: gender, sexual orientation, ethnicity and age; and socio-economic factors: UK birth/English reading ability, employment, housing, education and financial hardship. To assess non-adherence, participants were asked if in the past 3 months, they had missed ART for ≥2 days at a time. Virological suppression was defined as VL≤50 cps/mL. For each socio-economic factor, we calculated prevalence ratios using modified Poisson regression, first adjusting for demographic factors, then also for non-adherence.

**Results:**

A total of 2445 people fulfilled the inclusion criteria (80% male, 69% MSM, median age: 46 years, median CD4 count: 556 cells/mm^3^); 10% (234/2445) had VL>50 cps/mL. After adjusting for demographic factors, non-fluent English, not being employed, not home owning, education below university level and increasing financial hardship were each associated with higher prevalence of VL>50 cps/mL. Additional adjustment for non-adherence largely attenuated each association, but did not fully explain them (see Table 1). After adjustment for non-adherence and demographic factors, younger age was also associated with VL>50 cps/mL: for each additional 10 years an individual was 0.80 (95% CI 0.70–0.92) times as likely to have VL>50 cps/mL (p=0.0019). Adjusted prevalence ratios for VL>50cps/mL were 0.91 (0.62–1.34) for women and 1.25 (0.85–1.84) for non-MSM men versus MSM, and 1.29 (0.92–1.80) for white versus non-white people.

**Conclusions:**

Among people on ART in the United Kingdom, the proportion with detectable VL is low. Poorer socio-economic status is associated with increased probability of virological non-suppression. It is likely that much of this association is mediated through difficulties in taking ART. Emphasis should be put on aiding the adherence of people in these higher risk groups.

**Table 1:** Association between socio-economic factors and having VL > 50 copies/mL

		VL > 50 copies/mL		Adjusted for demographic factors^a^		Adjusted for demographic factors and non-adherence^b^	
							
Socio-economic factors		N (%)	p	Prevalence Ratio (95% CI)	p	Prevalence Ratio (95% CI)	p
Overall		234/2445 (10%)					
English reading ability	Born in the UK	114/1337 (9%)	0.0021~	1.00	0.0389	1.00	0.3058
	Fluent	73/841 (9%)		0.75 (0.54, 1.05)		0.85 (0.62, 1.17)	
	Not fluent	34/185 (18%)		1.28 (0.79, 2.05)		1.18 (0.73, 1.89)	
Employed	Yes	95/1316 (7%)	<.0001	1.00	<.0001	1.00	0.0057
	No	139/1129 (12%)		1.80 (1.39, 2.34)		1.44 (1.11, 1.87)	
Homeowner	Yes	44/861 (5%)	<.0001	1.00	<.0001	1.00	0.0007
	No	181/1537 (12%)		2.07 (1.48, 2.91)		1.74 (1.23, 2.45)	
University	Yes	72/986 (7%)	0.0051	1.00	0.0011	1.00	0.0214
	No	149/1390 (11%)		1.55 (1.18, 2.04)		1.36 (1.04, 1.78)	
Enough money for basic needs?	Always	66/1050 (6%)	<.0001~	1.00	0.0001	1.00	0.0224
Mostly	66/626 (11%)		1.68 (1.20, 2.34)		1.46 (1.05, 2.03)	
	Sometimes	48/423 (11%)		1.71 (1.18, 2.50)		1.29 (0.88, 1.89)	
	No	45/298 (15%)		2.32 (1.60, 3.37)		1.77 (1.21, 2.59)	

a Adjusted for age, gender, sexual orientation and ethnicity

b adjusted for demographics and ART non-adherence – self-reported ever missed ≥2 days.

~ Test for trend.

